# Supplementary material for: Early combination of albumin with crystalloids administration might not be beneficial for the survival of ischemic stroke patients with sepsis: A retrospective analysis from MIMIC‑IV database
Source: PLoS One. 2025 Dec 30;20(12):e0339687. doi: 10.1371/journal.pone.0339687 (PMC12752989; doi:10.1371/journal.pone.0339687)
Supplement: S1 File. — S1 Table. ICD-9 and ICD-10 codes for cerebral infarction. S2 Table. Baseline characteristics between the two groups after propensity score matching. S3 Table. Univariable COX analysis of the association between therapies and 90-day all-cause mortality before propensity score matching. S4 Table. Univariable COX analysis of the association between therapies and 90-day all-cause mortality after propensity score matching. S5 Table. Multivariable analysis of the association between therapies and outcomes after propensity score matching. S6 Table. Time-dependent Cox regression analysis of the association between therapies and outcomes before propensity score matching. S7 Table. Time-dependent Cox regression analysis of the association between therapies and outcomes after propensity score matching. S1 Fig. Forest plot for subgroup analysis of the relationship between combination therapy and 90-day all-cause mortality after propensity score matching. (DOCX) [file pone.0339687.s001.docx]

**Supporting information**

**S1 Table. ICD-9 and ICD-10 codes for cerebral infarction.**

| **ICD-9 codes** | 34660, 34661, 34662, 34663, 43301, 43311, 43321, 43331, 43381, 43391, 43401, 43411, 43491 |
| --- | --- |
| **ICD-10 codes** | G436, G4360, G43601, G43609, G4361, G43611, G43619, I63, I630, I6300, I6301, I63011, I63012, I63013, I63019, I6302, I6303, I63031, I63032, I63033, I63039, I6309, I631, I6310, I6311, I63111, I63112, I63113, I63119, I6312, I6313, I63131, I63132, I63133, I63139, I6319, I632, I6320, I6321, I63211, I63212, I63213, I63219, I6322, I6323, I63231, I63232, I63233, I63239, I6329, I633, I6330, I6331, I63311, I63312, I63313, I63319, I6332, I63321, I63322, I63323, I63329, I6333, I63331, I63332, I63333, I63339,I6334, I63341, I63342, I63343, I63349, I6339, I634, I6340, I6341, I63411, I63412, I63413, I63419, I6342, I63421, I63422, I63423, I63429, I6343, I63431, I63432, I63433, I63439, I6344, I63441, I63442, I63443, I63449, I6349, I635, I6350, I6351, I63511, I63512, I63513, I63519, I6352, I63521, I63522, I63523, I63529, I6353, I63531, I63532, I63533, I63539, I6354, I63541, I63542, I63543, I63549, I6359, I636, I638, I6381, I6389 |

**S2 Table.** **Baseline characteristics between the two groups after propensity score matching.**

| **Categories** | **Total population**  **n=320** | **Crystalloids alone**  **n=160** | **Combination**  **n=160** | ***P* value** |
| --- | --- | --- | --- | --- |
| Demographic |  |  |  |  |
| Age (years) (median, IQR) | 69.21; 59.54-78.70 | 71.02; 60.66-80.02 | 67.83; 58.55-77.37 | 0.080 |
| Female, n (%) | 183 (57.19) | 76 (47.50) | 67 (41.88) | 0.312 |
| Vital signs (median, IQR) |  |  |  |  |
| MAP (mmHg) | 77.18; 72.43-84.73 | 79.61; 73.15-87.30 | 75.69; 71.77-82.19 | 0.002 |
| Respiratory rate (breaths/min) | 18.68; 16.57-20.86 | 19.18; 17.34-20.94 | 17.86; 15.94-20.69 | 0.001 |
| Heart rate (beats/min) | 81.87; 72.96-91.23 | 83.14; 73.27-94.00 | 80.52; 72.47-90.40 | 0.477 |
| SpO2 (%) | 98.14; 96.84-99.03 | 97.85; 96.76-99.03 | 98.27; 96.99-99.05 | 0.224 |
| Comorbidities, n (%) |  |  |  |  |
| Hypertension | 249 (77.81) | 125 (78.13) | 124 (77.50) | 0.893 |
| Congestive heart failure | 106 (33.13) | 50 (31.25) | 56 (35.00) | 0.476 |
| Atrial fibrillation | 142 (44.38) | 67 (41.88) | 75 (46.88) | 0.368 |
| Diabetes | 120 (37.50) | 60 (37.50) | 60 (37.50) | 1.000 |
| Respiratory failure | 160 (50.00) | 75 (46.88) | 85 (53.13) | 0.264 |
| Chronic kidney disease | 73 (22.81) | 35 (21.88) | 38 (23.75) | 0.689 |
| Chronic pulmonary disease | 82 (25.63) | 39 (24.38) | 43 (26.88) | 0.609 |
| Hyperlipidemia | 162 (50.63) | 82 (51.25) | 80 (50) | 0.823 |
| Laboratory parameters (median, IQR) |  |  |  |  |
| WBC (10^9^/L) | 14.35; 10.78-18.80 | 14.20; 10.68-18.80 | 14.75; 10.95-19.00 | 0.369 |
| BUN (mg/dL) | 21.00; 15.00-29.00 | 22.50; 15.00-31.00 | 19.50; 15.00-28.00 | 0.053 |
| Serum creatinine (mg/dL) | 1.10; 0.80-1.50 | 1.10; 0.80-1.50 | 1.10; 0.80-1.50 | 0.285 |
| Platelet (10^9^/L) | 209.00; 165.00-275.00 | 224.50; 178.50-284.50 | 197.00; 150.00-261.00 | 0.220 |
| Hemoglobin (g/dL) | 11.55; 10.10-13.03 | 11.80; 10.20-13.40 | 11.25; 10.10-12.78 | 0.174 |
| Lactate (mmol/L) | 3.10; 2.10-5.00 | 2.80; 1.80-4.30 | 3.65; 2.40-6.08 | 0.001 |
| Severity scores (median, IQR) |  |  |  |  |
| SOFA | 3; 2-4 | 3; 2-4 | 3; 2-5 | 0.313 |
| GCS | 15; 12-15 | 15; 11-15 | 15; 13-15 | 0.225 |
| APS III | 46; 34-60 | 45; 33-59 | 46; 35-63 | 0.506 |
| Treatment, n (%) |  |  |  |  |
| Ventilation | 309 (96.56) | 153 (95.63) | 156 (97.50) | 0.357 |
| RRT | 34 (10.63) | 12 (7.50) | 22 (13.75) | 0.070 |
| Vasopressor ^a^ |  |  |  | <0.001 |
| None | 265 (82.81) | 150 (93.75) | 115 (71.88) |  |
| Within 24 hours | 36 (11.25) | 7 (4.38) | 29 (18.13) |  |
| After 24 hours | 19 (5.94) | 3 (1.88) | 16 (10.00) |  |
| RTPA | 3 (0.94) | 1 (0.63) | 2 (1.25) | 0.562 |
| Endovascular treatment | 24 (7.50) | 13 (8.13) | 11 (6.88) | 0.671 |
| Antibiotic-Carbapenems | 36 (11.25) | 14 (8.75) | 22 (13.75) | 0.157 |
| Antibiotic- Glycopeptide | 233 (72.81) | 106 (66.25) | 127 (79.38) | 0.008 |
| Antibiotic-β-lactams | 269 (84.06) | 135 (84.38) | 134 (83.75) | 0.879 |
| Antibiotic- Aminoglycosides | 25 (7.81) | 12 (7.50) | 13 (8.13) | 0.835 |
| Timing of antibiotic administration ^b^ |  |  |  | 0.219 |
| Within 24 hours | 163 (50.94) | 76 (47.50) | 87 (54.38) |  |
| After 24 hours | 157 (49.06) | 84 (52.50) | 73 (45.63) |  |
| Timing of crystalloid administration ^c^  (hours) (median, IQR) | 5.16; 2.21-31.56 | 6.28; 1.79-50.80 | 4.99; 2.68-20.35 | 0.551 |
| Volume of crystalloid administration  (mL) (median, IQR) | 2000; 1000-3000 | 1350; 800-2500 | 2600; 1500-3500 | <0.001 |

IQR, interquartile range; MAP, mean arterial blood pressure; WBC, white blood cell; BUN, blood urea nitrogen; SOFA, sequential organ failure assessment; GCS, Glasgow coma scale; APS III, acute physiology score-III; RRT, renal replacement therapy; RTPA, recombinant tissue plasminogen activator.

^a^ The timing of vasopressor use was defined as the time interval from the initiation of crystalloid infusion to the first administration of a vasopressor. Based on this, patients were categorized into three groups: no vasopressor use, early use (within 24 hours of crystalloid initiation), and late use (after 24 hours).

^b^ The timing of antibiotic administration was defined as the interval from ICU admission to the first dose of antibiotics. Patients were accordingly categorized into two groups: those who received antibiotics within 24 hours of ICU admission, and those who received them after 24 hours.

^c^ The timing of crystalloid administration was defined as the interval from ICU admission to the first administration of crystalloid fluid.

**S3 Table.** **Univariable COX analysis of the association between therapies and 90-day all-cause mortality before propensity score matching.**

| **Variables** | **HR** | **95% CI** | ***P* value** |
| --- | --- | --- | --- |
| Age | 1.00 | 0.98-1.01 | 0.598 |
| Gender | 0.86 | 0.62-1.20 | 0.376 |
| MAP | 1.01 | 0.99-1.02 | 0.478 |
| Respiratory rate | 1.03 | 0.98-1.07 | 0.223 |
| Hypertension | 1.05 | 0.71-1.55 | 0.814 |
| Congestive heart failure | 1.28 | 0.91-1.80 | 0.160 |
| Atrial fibrillation | 1.05 | 0.75-1.46 | 0.788 |
| Diabetes | 0.94 | 0.67-1.32 | 0.707 |
| Respiratory failure | 1.01 | 0.73-1.41 | 0.944 |
| Chronic kidney disease | 1.38 | 0.96-1.99 | 0.082 |
| Chronic pulmonary disease | 0.78 | 0.53-1.15 | 0.212 |
| Hyperlipidemia | 0.85 | 0.61-1.19 | 0.349 |
| WBC | 1.02 | 1.00-1.04 | 0.044 |
| BUN | 1.00 | 0.99-1.01 | 0.901 |
| Serum creatinine | 0.97 | 0.91-1.04 | 0.389 |
| Platelet | 1.00 | 1.00-1.00 | 0.401 |
| Hemoglobin | 1.02 | 0.95-1.09 | 0.597 |
| Lactate | 1.05 | 1.01-1.09 | 0.014 |
| SOFA | 1.03 | 0.94-1.12 | 0.548 |
| GCS | 0.97 | 0.93-1.01 | 0.175 |
| APS III | 1.01 | 1.01-1.02 | 0.001 |
| Ventilation | 0.70 | 0.33-1.49 | 0.355 |
| RRT | 1.08 | 0.66-1.77 | 0.763 |
| Vasopressor ^a^ |  |  |  |
| None | 1.0 (Reference) |  |  |
| Within 24 hours | 2.08 | 1.32-3.27 | 0.002 |
| After 24 hours | 1.88 | 1.07-3.28 | 0.028 |
| RTPA | 0.64 | 0.09-4.59 | 0.658 |
| Endovascular treatment | 0.61 | 0.33-1.13 | 0.117 |
| Antibiotic-Carbapenems | 1.25 | 0.75-2.11 | 0.395 |
| Antibiotic-Glycopeptide | 1.52 | 1.02-2.27 | 0.040 |
| Timing of antibiotic administration ^b^ |  |  |  |
| Within 24 hours | 1.0 (Reference) |  |  |
| After 24 hours | 1.46 | 1.05-2.02 | 0.025 |
| Volume of crystalloid administration | 1.00 | 1.00-1.00 | 0.689 |
| Timing of crystalloid administration ^c^ | 1.00 | 1.00-1.00 | 0.091 |
| Fluid therapy |  |  |  |
| Crystalloids alone | 1.0 (Reference) |  |  |
| Early combination | 1.28 | 0.84-1.93 | 0.246 |
| Non-early combination | 2.34 | 1.54-3.57 | <0.001 |

HR, hazard ratio; 95% CI, 95% confidence interval; MAP, mean arterial blood pressure; WBC, white blood cell; BUN, blood urea nitrogen; SOFA, sequential organ failure assessment; GCS, Glasgow coma scale; APS III, acute physiology score-III; RRT, renal replacement therapy; RTPA, recombinant tissue plasminogen activator.

^a^ The timing of vasopressor use was defined as the time interval from the initiation of crystalloid infusion to the first administration of a vasopressor. Based on this, patients were categorized into three groups: no vasopressor use, early use (within 24 hours of crystalloid initiation), and late use (after 24 hours).

^b^ The timing of antibiotic administration was defined as the interval from ICU admission to the first dose of antibiotics. Patients were accordingly categorized into two groups: those who received antibiotics within 24 hours of ICU admission, and those who received them after 24 hours.

^c^ The timing of crystalloid administration was defined as the interval from ICU admission to the first administration of crystalloid fluid.

**S4 Table.** **Univariable COX analysis of the association between therapies and 90-day all-cause mortality after propensity score matching.**

| **Variables** | **HR** | **95% CI** | ***P* value** |
| --- | --- | --- | --- |
| Age | 1.00 | 0.98-1.02 | 0.839 |
| Gender | 0.96 | 0.61-1.49 | 0.849 |
| MAP | 1.01 | 0.99-1.03 | 0.227 |
| Respiratory rate | 1.00 | 0.94-1.06 | 0.935 |
| Hypertension | 1.10 | 0.67-1.82 | 0.703 |
| Congestive heart failure | 1.15 | 0.74-1.79 | 0.539 |
| Atrial fibrillation | 1.16 | 0.75-1.77 | 0.507 |
| Diabetes | 0.84 | 0.54-1.30 | 0.424 |
| Respiratory failure | 0.92 | 0.59-1.42 | 0.694 |
| Chronic kidney disease | 1.64 | 1.05-2.56 | 0.030 |
| Chronic pulmonary disease | 1.02 | 0.62-1.67 | 0.940 |
| Hyperlipidemia | 0.90 | 0.58-1.37 | 0.613 |
| WBC | 1.02 | 0.99-1.05 | 0.180 |
| BUN | 1.00 | 0.99-1.01 | 0.592 |
| Serum creatinine | 1.02 | 0.92-1.14 | 0.722 |
| Platelet | 1.00 | 1.00-1.00 | 0.701 |
| Hemoglobin | 1.00 | 0.91-1.09 | 0.957 |
| Lactate | 1.03 | 0.98-1.08 | 0.308 |
| SOFA | 1.01 | 0.91-1.12 | 0.797 |
| GCS | 0.96 | 0.90-1.02 | 0.201 |
| APS III | 1.01 | 1.00-1.02 | 0.015 |
| Ventilation | 0.90 | 0.28-2.84 | 0.852 |
| RRT | 1.35 | 0.76-2.40 | 0.304 |
| Vasopressor ^a^ |  |  |  |
| None | 1.0 (Reference) |  |  |
| Within 24 hours | 1.54 | 0.87-2.72 | 0.135 |
| After 24 hours | 3.02 | 1.42-6.40 | 0.004 |
| RTPA | 1.02 | 0.99-1.05 | 0.266 |
| Endovascular treatment | 0.73 | 0.27-2.01 | 0.548 |
| Antibiotic-Carbapenems | 1.46 | 0.77-2.76 | 0.242 |
| Antibiotic-Glycopeptide | 1.44 | 0.84-2.49 | 0.189 |
| Timing of antibiotic administration ^b^ |  |  |  |
| Within 24 hours | 1.0 (Reference) |  |  |
| After 24 hours | 1.17 | 0.77-1.79 | 0.465 |
| Volume of crystalloid administration | 1.00 | 1.00-1.00 | 0.209 |
| Timing of crystalloid administration ^c^ | 1.00 | 1.00-1.00 | 0.857 |
| Fluid therapy |  |  |  |
| Crystalloids alone | 1.0 (Reference) |  |  |
| Early combination | 1.18 | 0.69-2.02 | 0.539 |
| Non-early combination | 2.72 | 1.65-4.50 | <0.001 |

HR, hazard ratio; 95% CI, 95% confidence interval; MAP, mean arterial blood pressure; WBC, white blood cell; BUN, blood urea nitrogen; SOFA, sequential organ failure assessment; GCS, Glasgow coma scale; APS III, acute physiology score-III; RRT, renal replacement therapy; RTPA, recombinant tissue plasminogen activator.

^a^ The timing of vasopressor use was defined as the time interval from the initiation of crystalloid infusion to the first administration of a vasopressor. Based on this, patients were categorized into three groups: no vasopressor use, early use (within 24 hours of crystalloid initiation), and late use (after 24 hours).

^b^ The timing of antibiotic administration was defined as the interval from ICU admission to the first dose of antibiotics. Patients were accordingly categorized into two groups: those who received antibiotics within 24 hours of ICU admission, and those who received them after 24 hours.

^c^ The timing of crystalloid administration was defined as the interval from ICU admission to the first administration of crystalloid fluid.

**S5 Table. Multivariable analysis of** **the association between therapies and outcomes after propensity score matching.**

| **Outcomes** | **Crystalloids** | **Combination** | ***P* value** | **Crystalloids** | **Early combination** | **Non-early combination** | ***P* value^a^** | ***P* value^b^** |
| --- | --- | --- | --- | --- | --- | --- | --- | --- |
| 90-day mortality |  |  |  |  |  |  |  |  |
| Unadjusted | 1.00 (reference) | 1.72 (1.12-2.65) | 0.013 | 1.00 (reference) | 1.18 (0.69-2.02) | 2.72 (1.65-4.50) | 0.539 | <0.001 |
| Model 1 | 1.00 (reference) | 1.73 (1.12-2.67) | 0.013 | 1.00 (reference) | 1.26 (0.70-2.27) | 3.40 (1.88-6.13) | 0.435 | <0.001 |
| Model 2 | 1.00 (reference) | 1.76 (1.13-2.76) | 0.013 | 1.00 (reference) | 1.17 (0.68-2.04) | 2.96 (1.73-5.05) | 0.569 | <0.001 |
| Model 3 | 1.00 (reference) | 1.87(1.14-3.08) | 0.013 | 1.00 (reference) | 1.23 (0.69-2.19) | 3.31 (1.84-5.96) | 0.489 | <0.001 |
| LOS Hospital |  |  |  |  |  |  |  |  |
| Unadjusted | 1.00 (reference) | 1.35 (1.09-1.68) | 0.006 | 1.00 (reference) | 1.11 (0.93-1.34) | 1.25 (1.02-1.55) | 0.246 | 0.034 |
| Model 1 | 1.00 (reference) | 1.32 (1.06-1.63) | 0.011 | 1.00 (reference) | 1.11 (0.93-1.32) | 1.20 (0.98-1.48) | 0.247 | 0.076 |
| Model 2 | 1.00 (reference) | 1.29 (1.05-1.60) | 0.016 | 1.00 (reference) | 1.07 (0.89-1.28) | 1.19 (0.98-1.47) | 0.461 | 0.088 |
| Model 3 | 1.00 (reference) | 1.16 (0.95-1.43) | 0.145 | 1.00 (reference) | 1.04 (0.88-1.24) | 1.15 (0.95-1.39) | 0.613 | 0.164 |
| LOS ICU |  |  |  |  |  |  |  |  |
| Unadjusted | 1.00 (reference) | 1.35 (1.09-1.68) | 0.006 | 1.00 (reference) | 1.19 (0.93-1.53) | 1.61 (1.21-2.16) | 0.183 | 0.002 |
| Model 1 | 1.00 (reference) | 1.32 (1.06-1.63) | 0.011 | 1.00 (reference) | 1.18 (0.92-1.52) | 1.53 (1.16-2.06) | 0.196 | 0.004 |
| Model 2 | 1.00 (reference) | 1.29 (1.05-1.06) | 0.016 | 1.00 (reference) | 1.12 (0.88-1.45) | 1.53 (1.16-2;03) | 0.345 | 0.003 |
| Model 3 | 1.00 (reference) | 1.16 (0.95-1.43) | 0.145 | 1.00 (reference) | 1.01 (0.80-1.28) | 1.37 (1.06-1.79) | 0.936 | 0.019 |

Data are presented as the HR (95%CI). Model 1 was adjusted for age and sex. Model 2: Model 1 plus lactate, APS III score, and WBC. Model 3: Model 2 plus vasopressor use, timing of antibiotic administration, congestive heart failure, chronic kidney disease, chronic pulmonary disease, and glycopeptide use.

*P* value^a^ was used to indicate the differences between the crystalloids group and the early combination group. *P* value^b^ was used to indicate the differences between the crystalloids group and the non-early combination group.

**S6 Table. Time-dependent Cox regression analysis of the association between therapies and outcomes before propensity score matching.**

|  | **Comparison** | **Time Period** | **HR (95%CI)** | ***P* value** |
| --- | --- | --- | --- | --- |
| **Unadjusted** | Crystalloids vs Early combination | Early phases | 0.93 (0.56-1.56) | 0.791 |
|  | Crystalloids vs Non-early combination | Early phases | 1.14 (0.66-1.98) | 0.646 |
|  | Crystalloids vs Early combination | Late phases | 1.76 (0.88-3.54) | 0.151 |
|  | Crystalloids vs Non-early combination | Late phases | 5.00 (2.55-9.81) | <0.001 |
| **Demographics adjusted** | Crystalloids vs Early combination | Early phases | 0.94 (0.56-1.59) | 0.829 |
|  | Crystalloids vs Non-early combination | Early phases | 1.12 (0.65-1.96) | 0.678 |
|  | Crystalloids vs Early combination | Late phases | 1.79 (0.89-3.62) | 0.148 |
|  | Crystalloids vs Non-early combination | Late phases | 4.88 (2.48-9.60) | <0.001 |
| **Fully adjusted** | Crystalloids vs Early combination | Early phases | 0.69 (0.39-1.22) | 0.207 |
|  | Crystalloids vs Non-early combination | Early phases | 1.02 (0.54-1.91) | 0.953 |
|  | Crystalloids vs Early combination | Late phases | 1.61 (0.77-3.39) | 0.065 |
|  | Crystalloids vs Non-early combination | Late phases | 6.18 (2.86-13.35) | <0.001 |

Demographic adjustment refers to adjustment for age and sex. Fully adjustment refers to adjustment for age, sex, lactate, APS III score, WBC, vasopressor use, timing of antibiotic administration, congestive heart failure, chronic kidney disease, chronic pulmonary disease, and glycopeptide use.

Early phases: 0-28 days; Late phases: 29-90 days.

**S7 Table. Time-dependent Cox regression analysis of the association between therapies and outcomes after propensity score matching.**

|  | **Comparison** | **Time Period** | **HR (95%CI)** | ***P* value** |
| --- | --- | --- | --- | --- |
| **Unadjusted** | Crystalloids vs Early combination | Early phases | 1.11 (0.54-2.26) | 0.781 |
|  | Crystalloids vs Non-early combination | Early phases | 1.61 (0.82-3.20) | 0.168 |
|  | Crystalloids vs Early combination | Late phases | 1.05 (0.47-2.36) | 0.924 |
|  | Crystalloids vs Non-early combination | Late phases | 3.87 (1.86-8.06) | 0.088 |
| **Demographics adjusted** | Crystalloids vs Early combination | Early phases | 1.12 (0.55-2.29) | 0.759 |
|  | Crystalloids vs Non-early combination | Early phases | 1.64 (0.82-3.26) | 0.159 |
|  | Crystalloids vs Early combination | Late phases | 1.06 (0.47-2.39) | 0.926 |
|  | Crystalloids vs Non-early combination | Late phases | 4.08 (1.91-8.71) | 0.077 |
| **Fully adjusted** | Crystalloids vs Early combination | Early phases | 0.99 (0.46-2.14) | 0.987 |
|  | Crystalloids vs Non-early combination | Early phases | 1.55 (0.74-3.24) | 0.247 |
|  | Crystalloids vs Early combination | Late phases | 1.27 (0.54-2.97) | 0.671 |
|  | Crystalloids vs Non-early combination | Late phases | 5.73 (2.50-13.17) | 0.015 |

Demographic adjustment refers to adjustment for age and sex. Fully adjustment refers to adjustment for age, sex, lactate, APS III score, WBC, vasopressor use, timing of antibiotic administration, congestive heart failure, chronic kidney disease, chronic pulmonary disease, and glycopeptide use.

Early phases: 0-28 days; Late phases: 29-90 days.


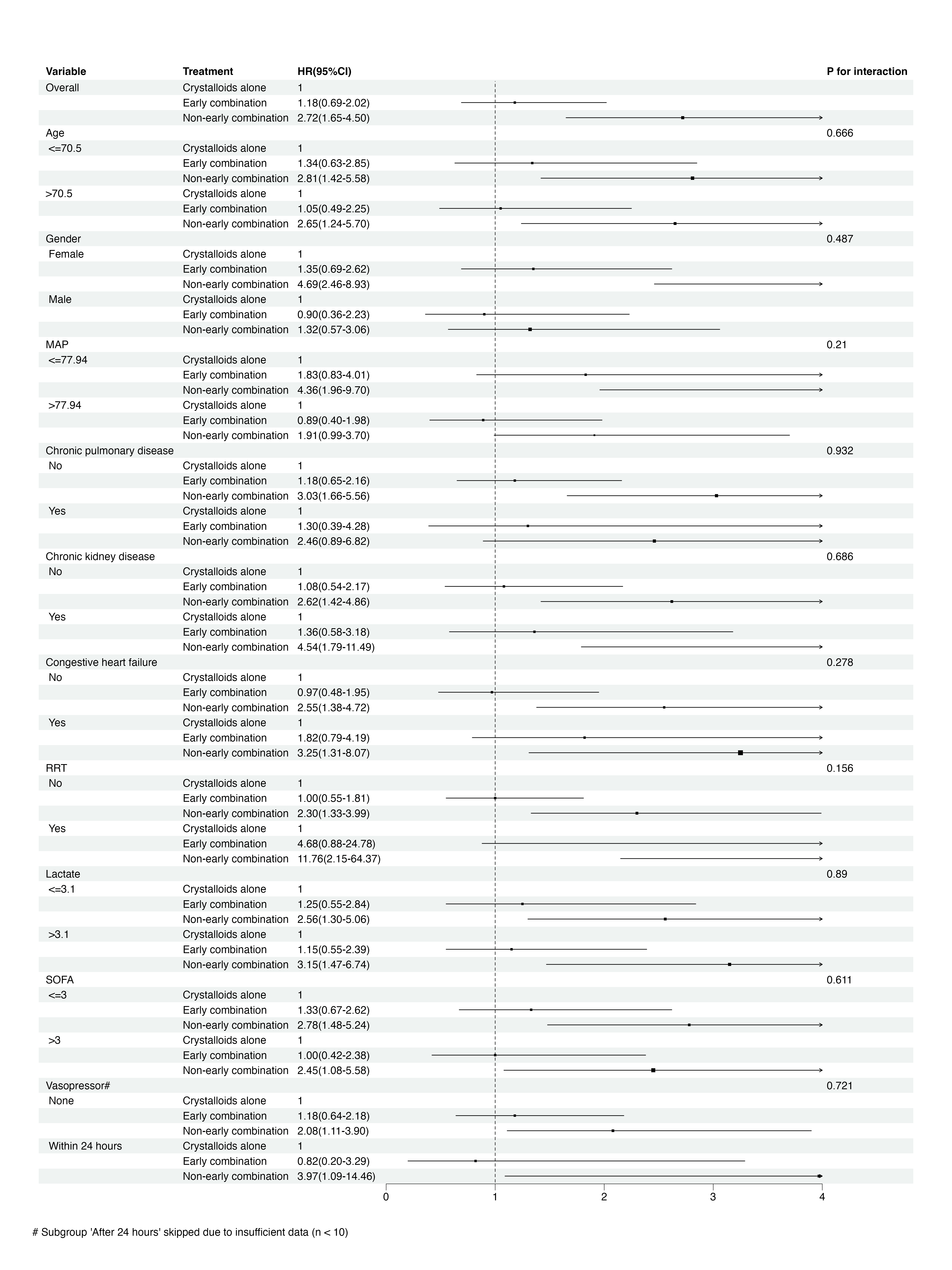


**S1 Fig. Forest plot for subgroup analysis of the relationship between combination therapy and 90-days all-cause mortality after propensity score matching.** F, female; M, male; HR, hazard ratio; CI, confidential interval.
