# Supplementary material for: Early combination of albumin with crystalloids administration might not be beneficial for the survival of ischemic stroke patients with sepsis: A retrospective analysis from MIMIC‑IV database
Source: PLoS One. 2025 Dec 30;20(12):e0339687. doi: 10.1371/journal.pone.0339687 (PMC12752989; doi:10.1371/journal.pone.0339687)
Supplement: Attachment — Submitted filename: Response_to_Reviewers_auresp_2.docx [file pone.0339687.s004.docx]

**Dear Editor,**

Manuscript number: PONE-D-25-09820R1

Title: Early combination of albumin with crystalloids administration might not be beneficial for the survival of ischemic stroke patients with sepsis: a retrospective analysis from MIMIC‑IV database

Authors: Yuming Teng, Min Li, Mo Yuan

Thank you for giving us an opportunity to revise our manuscript. We have revised the manuscript carefully according to the reviewers’ comments and recommendations. The response to each question or comment of reviewers was listed point-by-point in the attached pages. We hope that the revised form is suitable for publication in your journal.

Thank you for your consideration.

Yours sincerely,

Mo Yuan

School of Medicine, Yunnan University, Kunming, China, Dong wai huan South Road, University Town, Chenggong District, Kunming City, Yunnan Province 650000, China.

Tel: 0086-871-65033825

E-mail: ym@ynu.edu.cn

**Answers to editor**

*Major comments*

1. *Line 73-75: Authors training course completion detail not required in the manuscript. Remove “To access this database, ………………………………………….. and passed the examination (ID: 57478133).*

Answer: Thank you for your suggestions. I have deleted the relevant content as per your request.

2. *Please justify with similar studies with respect to the exemption for ethical approval.*

Answer: Thank you for your strict and rigorous requirements. In accordance with your requirements, we have further justified the rationale for ethical approval exemption：

“MIMIC-IV is a public clinical database co-developed by Beth Israel Deaconess Medical Center (BIDMC) and MIT, sourced from BIDMC’s deidentified routine care data. Its creation and public sharing received an ethical waiver from BIDMC’s Institutional Review Board, which granted informed consent exemption and approved data sharing [1]. Access is restricted to investigators who have completed required ethics training and have signed a Data Use Agreement [2]. In compliance with these requirements, our research team has fulfilled all access prerequisites. Therefore, this retrospective study meets the exemption criteria for ethical review, and this practice has an established precedent in peer-reviewed studies utilizing the MIMIC-IV database [3, 4].”

We have supplemented the aforementioned justifications along with citations of References [1], [2], [3], and [4] to the "Ethics Statement" section under the "Methods" part of the manuscript (see Page 6, Lines 84-91 and Page 7, Lines 92-93).

reference：

1.	Johnson A, Bulgarelli L, Pollard T, Horng S, Celi LA, Mark R. Mimic-iv. PhysioNet; doi:10.13026/6MM1-EK67

2.	Johnson AEW, Bulgarelli L, Shen L, Gayles A, Shammout A, Horng S, et al. MIMIC-IV, a freely accessible electronic health record dataset. Sci Data. 2023;10: 1. doi:10.1038/s41597-022-01899-x

3.	Zhou S, Zeng Z, Wei H, Sha T, An S. Early combination of albumin with crystalloids administration might be beneficial for the survival of septic patients: A retrospective analysis from MIMIC-IV database. Ann Intensive Care. 2021;11: 42. doi:10.1186/s13613-021-00830-8

4.	Li X-Y, Chen W-S, Qu Z-K, Chen J-G, Li L, Li S-N, et al. Early use of albumin may increase the risk of sepsis-associated acute kidney injury in sepsis patients: A target trial emulation. Mil Med Res. 2025;12: 51. doi:10.1186/s40779-025-00641-z

3. *Line 89-90: Add reference for the sentence "Access is restricted to investigators who have completed required ethics training and have signed a Data Use Agreement.".*

**Answer:** Thank you for your constructive comments. The relevant references have been added as requested.

4. *Add figures with good quality.*

**Answer:** Concerning the figure-related issue, we have repeatedly verified that the submitted images are at a resolution of 600 DPI. Furthermore, when clicking the link "Click here to access/download; Figure; Fig.#-plosone" in the preview PDF, the downloaded images remain extremely clear even when enlarged. Therefore, we kindly request that you verify the matter again. Should any issues arise, please feel free to contact us promptly. Thank you for your assistance.
